# Comparison between Refractive Outcome of Primary Piggyback Intraocular Lens versus Secondary Lens Iris Claw Lens in Posterior Microphthalmos

**DOI:** 10.1155/2019/1356982

**Published:** 2019-02-14

**Authors:** Abdelhamid Elhofi, Hany Ahmed Helaly, Amr Said

**Affiliations:** Ophthalmology Department, Faculty of Medicine, Alexandria University, Alexandria, Egypt

## Abstract

**Purpose:**

To compare the refractive outcome of 2 different methods of intraocular lens implantation in cases of posterior microphthalmos, primary piggyback IOLs versus secondary iris claw lenses.

**Methods:**

This study was a retrospective interventional comparative study that included 60 eyes of 30 patients. The included patients had bilateral microphthalmos with high axial hyperopia and had undergone a lens-based surgical procedure for hyperopia correction. The included patients were equally divided into two groups. The first group had undergone refractive lens exchange (RLE) with primary piggyback IOL implantation. The second group undergone RLE with maximum available IOL power implanted followed by a secondary implantation of Artisan iris-fixated IOL (Ophtec B.V., Groningen, the Netherlands).

**Results:**

The 2 groups were highly comparable to each other regarding the mean age, axial length (AL), manifest refraction (MR), and *K* readings. Postoperatively, there was a statistically significant difference between the 2 groups regarding the manifest refraction spherical equivalent (MRSE), mean absolute error (MAE), and uncorrected distance visual acuity (UDVA). There was no significant difference between the 2 groups regarding the CDVA. At 36 months, 20% and 73% of the eyes were within ±0.5 D of intended refraction at 36 months in 1ry piggyback and 2ry Artisan groups, respectively. Fifty-three percent and 93% of the eyes were within ±1.0 D of intended refraction at 36 months in 1ry piggyback and 2ry Artisan groups, respectively (*p*=0.001).

**Conclusion:**

Secondary procedure with implantation of iris-fixated intraocular lens yielded very good results for treatment of axial hyperopia in cases of posterior microphthalmos. The primary piggyback IOL showed less satisfactory results with cases of under correction and the possible complication of interlenticular opacification. Both groups showed good safety parameters.

## 1. Introduction

Microphthalmos is a developmental ocular disorder arising from halted growth of the ocular tissues [1], resulting in a short axial length at least two standard deviations below the normal for the patient's age [2]. It includes various phenotype subsets as nanophthalmos and posterior microphthalmos in which the reduced axial length is secondary to just posterior segment foreshortening, with a relatively normal anterior segment [3]. The most commonly encountered clinical characteristics for this entity are axial hyperopia [4] and papillomacular folds [5]. Having normal corneal diameter and anterior chamber depth, posterior microphthalmos is commonly overlooked during routine pediatric eye assessment till patients develop obvious symptoms as hyperopia or even strabismus [6].

Cataract surgery in eyes with posterior microphthalmos presents a unique set of challenges not only due to the high rate of postoperative choroidal effusion [7] but also due to decreased predictability of standard methods for intraocular lens power calculation [8]. There is currently no definite global consensus regarding the ideal intraocular lens (IOL) power calculation formula in small eyes as those with microphthalmos, but some reports have suggested that the Haigis, Hoffer Q, or Holladay II formula may show some superiority for short eyes [9, 10].

The concept of using two piggyback IOLs has been investigated in eyes with short axial lengths with promising results [11–13] since the first attempt of Gayton and Sanders in a case of cataract and microphthalmos, in which the calculated IOL power was +46 diopters [11]. However, refractive surprises may still take place. Using a secondary Artisan iris-fixated phakic IOL (Ophtec B.V., Groningen, the Netherlands) [14, 15] depending on actual postoperative refraction rather than prediction may offer a solution to this dilemma, although the literature is scant about this technique [16].

The aim of the current study was to compare the refractive outcome of 2 different methods of intraocular lens implantation in cases of posterior microphthalmos, primary piggyback IOLs and secondary iris claw lenses implanted as a secondary procedure depending on actual postoperative refraction.

## 2. Subjects and Methods

This study was a retrospective interventional comparative study that included 60 eyes of 30 patients. The included patients had bilateral microphthalmos with high axial hyperopia and had undergone a lens-based surgical procedure for hyperopia correction. The included patients were equally divided into two groups. The first group (30 eyes of 15 patients) had undergone refractive lens exchange (RLE) with primary piggyback IOL implantation. The second group (30 eyes of 15 patients) had undergone RLE with maximum available IOL power implanted followed by a secondary implantation of Artisan iris-fixated IOL (Ophtec B.V., Groningen, the Netherlands). The inclusion criteria were having posterior microphthalmos defined as short axial length two standard deviations below the normal for the patient's age with normal corneal size and anterior chamber depth (ACD) and having complete records for 3 years follow-up postoperative. For secondary Artisan iris-fixated IOL, ACD should be > 2.8 mm. Patients were excluded if they had intraoperative complications, e.g., primary aphakia, incomplete data records, nanophthalmic eyes, microphthalmos with a corneal diameter less than 9 mm, microphthalmos with anterior segment dysgenesis syndrome, and posterior or combined types of persistent hyperplastic primary vitreous. Cases of macular scars and glaucoma were also excluded. Presence of papillomacular folds was not considered an exclusion criterion as it is commonly encountered in cases of posterior microphthalmos.

The current study was approved by the local ethics committee of the faculty medicine, Alexandria University, Egypt. Tenets of the Declaration of Helsinki were followed. All patients signed an informed consent explaining the procedure and possible complications.

Preoperative complete ophthalmic examinations were conducted including measuring corneal diameter and axial length, fundus examination for posterior segment abnormalities, and measuring intraocular pressure. Also, screening for systemic disorders was conducted. IOL power calculation was performed in all cases using optical biometry (IOL-master 500, Carl Zeiss, Germany) with the Haigis formula. Records of the patients were revised, and all preoperative, operative, and postoperative data were recorded. All patients were operated upon by the same surgeon (A.H.) with a reproducible technique.

### 2.1. Surgical Technique

All included cases had undergone a refractive lens exchange with corneal incisions created using a 2.4 mm keratome for main incision placed in all cases on the steep topographic axis as determined using corneal topography and 1.2 mm blade for the side ports. The anterior chamber was filled with viscoelastic material. The continuous curvilinear capsulorhexis (CCC) was created with a capsulorhexis forceps. Lens aspiration was performed using minimum or no phaco power according to the nuclear density. Infinity® Phacoemulsification System (Alcon, USA) was used in all cases. The procedure was followed by IOL implantation of the maximum power available in the capsular bag after removal of the lens cortex: Tecnis-1 aspheric IOL (Advanced Medical Optics (AMO)) using its injector provided by the company. Careful removal of the viscoelastic material from the anterior chamber was carried out in all cases followed by careful stromal hydration of all corneal wounds.

The first group had primary implantation of piggyback silicon 3-piece IOL in the sulcus (Tecnis 3-piece, Advanced Medical Optics (AMO)). The power of the piggyback IOL was calculated by subtracting the power of the already implanted IOL from the calculated IOL power and subtracting 0.5 D due to the change in the effective lens position because of sulcus placement. The second group had undergone another procedure after approximately 2 months in the form of a secondary implantation of Artisan iris-fixated IOL. The Artisan lens aphakia model 205 is a monofocal one-piece convex-concave PMMA IOL with an 8.5 mm length, a 1.04 mm maximum height, and a 5.0 mm optical zone. The haptics have grooves in which the iris can be enclavated. The available IOL powers ranged from +2.0 to +30.0 D. The calculation of the IOL power was done based on the actual residual refractive error of the eye after RLE. The power calculations were based on the “Van der Heijde” formula. The 2ry Artisan implantation was done using the same standardized technique of the phakic model. Two small corneal paracenteses at 3 and 9o'clock were performed, followed by a 5.5 mm corneoscleral tunnel at the 12 o'clock site. Insertion of the Artisan lens under the protection of a cohesive viscoelastic material in the anterior chamber was done followed by rotating the lens such that the haptics were opposing 3 and 9 o'clock positions. The lens was then held by the fixation forceps through the corneoscleral tunnel, and the midperipheral iris was enclavated into the haptics using an enclavation needle. The wound was closed with 10-0 nylon suture, and the cohesive viscoelastic was washed. Suture removal was done 2 months postoperatively.

Postoperative antibiotic and steroid eye drops were prescribed for one month. Patients were followed-up at day 1, week 1, and months 1, 3, 6, 12, 18, 24, and 36. The main outcome parameters were manifest refraction, mean absolute error (MAE) of prediction, uncorrected distance visual acuity (UDVA), corrected distance visual acuity (CDVA), Snellen lines loss of CDV, and predictability with percentage of eyes within 0.50 D and 1.0 D of intended correction. The MAE is calculated by the mean absolute value of the difference between intended and actual postoperative refraction.

Data analysis was performed using the software SPSS for Windows version 20.0 (SPSS Inc., Chicago, USA). Quantitative data were described using range, mean, and standard deviation. The Kolmogorov–Smirnov test was used for checking the normality of distribution. The independent sample *t*-test was used to compare means of different samples. The paired *t*-test was used for comparisons between means of the preoperative and postoperative data of the same eyes. The chi-square test was used to compare between different percentages. Pearson correlation was used to correlate between different variables. Differences were considered statistically significant when the associated *p* value was less than 0.05. Standard figures for reporting the outcomes in refractive surgery, according to the Waring Protocol and its modification, were used for displaying and summarizing the refractive outcomes of this study for each group postoperatively [17, 18].

## 3. Results

The current study was a retrospective comparative analysis of the records of 60 eyes of 30 patients with bilateral posterior microphthalmos.  Table 1 shows the demographic and preoperative characteristics of the included patients. There was no statistically significant difference between the 2 groups regarding the preoperative characteristics. The 2 groups were highly comparable to each other regarding the mean age, axial length (AL), manifest refraction (MR), and *K* readings. None of the eyes reached 20/20 of CDVA, and the worst CDVA was 20/125. The mean corneal diameter, central corneal thickness (CCT), and ACD were within normal range for both groups. Using Pearson correlation, the AL showed strong negative correlation with *K* readings (*r* = −0.83, *p*=0.001) and good positive correlation with corneal diameter (*r* = 0.60, *p*=0.001). Subgroup analysis of the right eye or the left eye alone did not show any different results.

Table 2 shows the visual acuity (UDVA and CDVA), MAE, and manifest refraction along the postoperative follow-up period. There was a statistically significant difference between the 2 groups regarding the manifest refraction spherical equivalent (MRSE), MAE, and UDVA. There was no significant difference between the 2 groups regarding the CDVA. Using paired *t*-test to compare mean UDVA, CDVA, MAE, and MRSE at 1 month and 36 months, there was no statistically significant difference. The mean CDVA at 36 months improved in comparison with preoperative levels for both groups, but this improvement was not statistically significant. The UDVA, MAE, and MRSE improved greatly from preoperative levels at 1 month postoperatively, and this improvement remained stable over the whole follow-up period. At 24 months, mean CDVA of 1ry piggyback group decreased but returned to its levels at 36 months. This difference was not statistically significant. There was a good negative correlation between the axial length and MAE (*r* = - 0.68, *p*=0.03 and *r* = −0.53, *p*=0.04 for 1ry piggyback and 2ry Artisan groups, respectively). There was no significant correlation between UDVA and CDVA in both groups.

**Figure 1:**
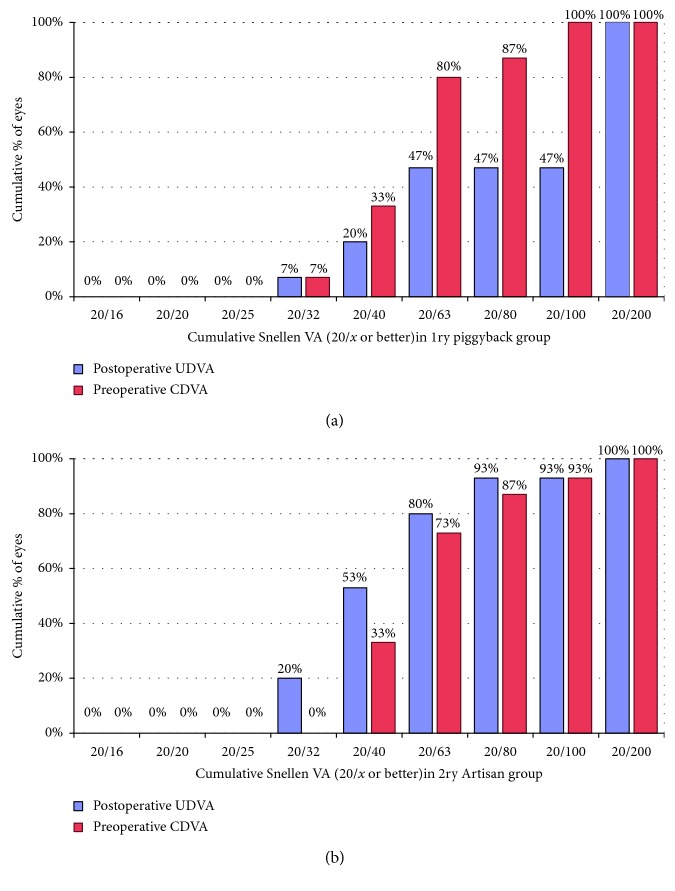
Cumulative Snellen visual acuity in 1ry piggyback (a) and 2ry Artisan (b) groups at 36 months.

**Figure 2:**
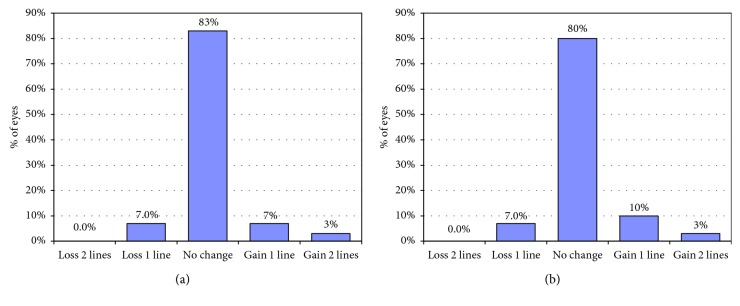
Changes in Snellen line in corrected distance visual acuity in 1ry piggyback group (a) and 2ry Artisan group (b) at 36 months.

Figure 1 shows the cumulative Snellen visual acuity in both groups at 36 months. The mean preoperative CDVA was significantly better than the mean UDVA at 36 months for the 1ry piggyback group (*p*=0.003). The mean UDVA at 36 months for the 2ry Artisan group was slightly better than the mean preoperative CDVA (0.39 ± 0.17 D vs. 0.45 ± 0.16 D). However, the difference was not statistically significant (*p*=0.490). The efficacy index at 36 months was 0.63 and 1.14 for 1ry piggyback and 2ry iris claw lens groups, respectively. The safety index at 36 months was 1.04 and 1.13 for 1ry piggyback and 2ry Artisan groups, respectively. Only 2 eyes from each group lost 1 line of Snellen visual acuity (Figure 2). No significant intra- or postoperative complications were recorded. Interlenticular opacification (ILO) was detected in 4 eyes of the 1ry piggyback group at end of the 2nd year of follow-up and was managed by the YAG laser. One eye of each group gained 2 lines of Snellen visual acuity. Using the chi-square test, the percentages of eyes that showed a change (either loss of gain) of Snellen visual acuity at 36 months were not statistically significantly different between the 2 groups (*p*=0.90).

**Figure 3:**
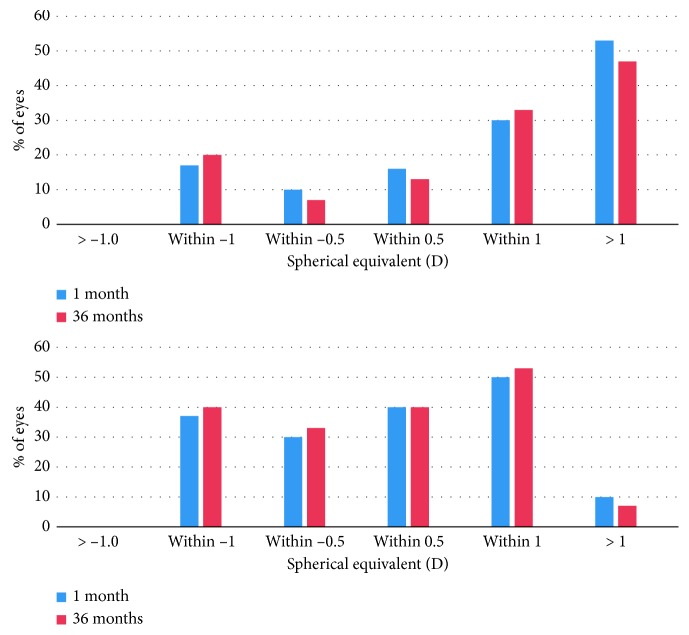
Distribution of postoperative spherical equivalent (predictability) among 1ry piggyback group (above) and 2ry Artisan group (below) at 1 month and 36 months. (a) Spherical equivalent (D): 1ry piggyback. (b) Spherical equivalent (D): 2ry Artisan.

**Figure 4:**
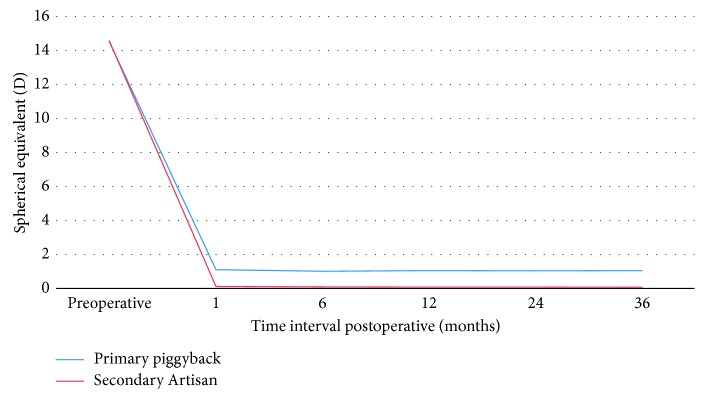
Stability of manifest refraction spherical equivalent over time in 1ry piggyback group and 2ry Artisan group.

**Figure 5:**
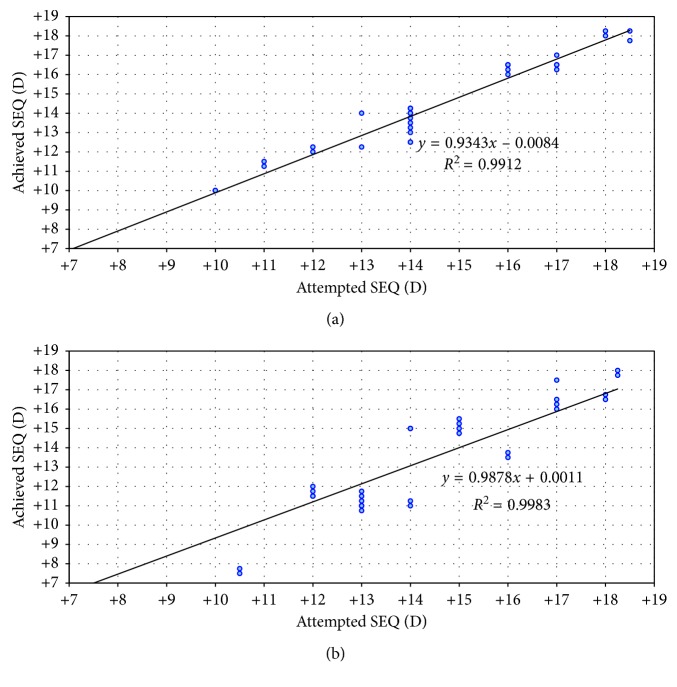
Attempted versus achieved manifest refraction spherical equivalent (SEQ) in 1ry piggyback group (a) and 2ry Artisan group (b) at 36 months.

At 36 months, 20% and 73% of the eyes were within ±0.5 D of intended refraction at 36 months in 1ry piggyback and 2ry Artisan groups, respectively. Fifty-three percent and 93 % of the eyes were within ±1.0 D of intended refraction at 36 months in 1ry piggyback and 2ry Artisan groups, respectively (Figure 3). Using the chi-square test, this difference was statistically significant (*p*=0.001). Regarding stability, both groups showed significant improvement of MRSE from preoperative levels at month 1 and remained stable along the whole follow-up period (Figure 4).  Figure 5 shows the attempted versus achieved MRSE in both groups at 36 months postoperatively. The dots above the trend line represent over correction with myopic residual error, and the dots below the trend line represent under correction with hyperopic residual error.

## 4. Discussion

Although a rare ocular disorder, posterior microphthalmos represents a problematic entity in modern lens-based surgery due to surgical difficulties and a higher incidence of postoperative complications as residual hyperopia, choroidal effusion, and the presence of ametropic amblyopia [1].

The modern cataract refractive surgery provides more satisfactory postoperative refractive outcome than ever with more than 55% of eyes within 0.5D and 85% eyes were within 1.0 diopter of the calculated target refraction according to the United Kingdom National Health System reports [19]. However, eyes with short axial lengths than usual may provide a refractive challenge. Haigis and Hoffer Q formulas were studied, revealing good outcomes in short eyes [20]. However, another issue appeared; there was a limited availability of high-power IOLs. To solve this dilemma, primary piggyback IOL has been tried in short eyes. The choice of the proper power depends on prediction. Several drawbacks exist regarding this option as the risk of interlenticular opacification (ILO), pupillary block, and pigment dispersion [21].

Another alternative was to implant another IOL as a secondary procedure depending on the actual postoperative refraction. Several alternatives exist for secondary IOL including iris claw lenses. The use of these lenses needs at least 2.8 mm depth of the anterior chamber and cannot be applied in patients with uveitis, glaucoma, or structural iris abnormalities [16].

To our knowledge, this is the first case series comparing these cases with the conventional treatment using piggyback IOLs. Most previous studies dealt with iris claw lenses as a solution for aphakic hypermetropia in the absence of adequate capsular bag support [22] or as a refractive phakic IOL [14].

Regarding the included eyes in the current study, the preoperative demographic and biometric data were comparable with no significant differences between the 2 groups. All eyes had high axial hyperopia with short axial length. The IOL power calculation yielded high positive lens power beyond the highest available IOL powers. So, a primary hydrophobic IOL with a power of +30 D was implanted in the bag with the residual error either treated by a 1ry piggyback IOL or a 2ry Artisan iris-fixated IOL. The Artisan lens used was the aphakic model because of the need for the availability of a plus-power IOL. The larger wound needed (5.5 mm) with 10-0 suture yielded higher astigmatism but due to the coupling effect, there was no effect on the overall MRSE. All the included cases were bilateral, having some degree of amblyopia in the eye of higher error. As shown in the preoperative data, the included eyes had within normal range sized corneas with normal depth anterior chambers. However, the corneas were steep, showing a negative correlation with the axial length.

The 2ry Artisan iris-fixated IOL group showed marked superiority regarding the postoperative MRSE, mean MAE, and consequently UDVA. In the 1ry piggyback group, there were many cases of under correction with hyperopic residual errors up to 3 D. Depending on the actual residual refractive error proved to greatly improve the refractive outcome. Both groups showed good level of safety regarding the loss of lines of Snellen visual acuity. Also, the mean postoperative CDVA in both groups was comparable despite the difference in the mean MRSE. The 2ry iris claw lens group showed excellent predictability with 93% of cases within ±1 D of intended refraction which is a very good result in such difficult cases.

The literature lacks reports of the use of iris claw lenses for short eyes after primary implantation of IOLs. An earlier report of piggyback IOLs in forty-three eyes of 32 patients (aged 19–87 years; median, 69 years) with nanophthalmos showed a median postoperative refraction at 6 months of +1.38 D (range, −2.00 to +12.50 D). The median difference between target refraction and achieved postoperative refraction was +0.84 D (range, −2.61 to +4.33 D). 43.9% of cases were within ±1 D of target refraction and 56.1% were not within ±1 D of target refraction [23].

It has to be mentioned that iris claw lens has some disadvantages. It is more prone to result in chronic uveitis, endothelial cell loss, and iris damage including ovalization and atrophy. The lens itself tends to be more expensive option than a secondary piggyback IOL. Exchanging the iris claw lens is theoretically more difficult than exchanging a secondary piggyback IOL. Early and late lens dislocation of the iris can occur as well. However, no series lens related complications were encountered in our case series.

Our study has some limitations. The retrospective nature may represent a source of selection bias. Lack of randomization or matching may also be a weak point. Lucky for us, there was no statistically significant difference between the 2 groups regarding the preoperative characteristics. As mentioned above, the 2 groups were highly comparable to each other. The rare nature of the disease has made matching not applicable. Other measures for visual function rather than visual acuity as contrast sensitivity and high-order aberrations were not assessed in our series.

In conclusion, secondary procedure with implantation of iris-fixated intraocular lens yielded very good results for treatment of axial hyperopia in cases of posterior microphthalmos. The primary piggyback IOL showed less satisfactory results with cases of under correction and the possible complication of interlenticular opacification. Both groups showed good safety parameters.

## Figures and Tables

**Table 1:** Preoperative characteristics of the included eyes (60 eyes of 30 patients divided equally into two groups).

	1ry piggyback (*n* = 30) mean + SD range	2ry Artisan (*n* = 30) mean + SD range	*p* value
Male : female	6 : 9	8 : 7	0.464^a^
Age (years)	22.7 + 4.1(18–29)	22.2 + 3.5 (19–30)	0.781^b^
MRSE (D)	14.52 + 2.3 (10.5–18.25)	14.57 + 2.5 (10–18.5)	0.925^b^
CDVA (log MAR)	0.43 + 0.15 (0.2–0.7)	0.45 + 0.16 (0.3–0.8)	0.638^b^
AL (mm)	16.6 + 1.2 (14.9–19.3)	16.7 + 1.2 (15.1–19.0)	0.854^b^
CCT (microns)	523.1 + 22.6 (499–551)	528.4 + 21.1 (502–559)	0.545^b^
*K* readings (D)	47.5 + 2.4 (43.0–52.0)	47.3 + 2.5(43.5–51.0)	0.635^b^
Corneal diameter	11.95 + 0.30 (11.50–12.20)	11.85 + 0.28 (11.40–12.25)	0.565^b^
ACD (mm)	2.95 ± 0.11 (2.82–3.3)	3.01 ± 0.21 (2.85–3.4)	0.487^b^

MRSE: manifest refraction spherical equivalent; CDVA: corrected distance visual acuity; AL: axial length; CCT: central corneal thickness; *K*: keratometry; ACD: anterior chamber depth. ^a^Chi-square test; ^b^independent sample *t*-test.

**Table 2:** Visual acuity and refraction along the postoperative follow-up period.

Mean + SD range	1 month	6 months	12 months	24 months	36 months
1ry piggyback: UDVA (logMAR)	0.73 + 0.34 (0.3 to 1.0)	0.70 + 0.32 (0.3 to 1.0)	0.71 + 0.29 (0.3 to 1.0)	0.68 + 0.30 (0.2 to 1.0)	0.69 + 0.30 (0.2 to 1.0)
2ry Artisan: UDVA (logMAR)	0.43 + 0.22 (0.3 to 0.9)	0.41 + 0.19 (0.2 to 0.9)	0.39 + 0.16 (0.2 to 0.8)	0.40 + 0.18 (0.2 to 0.8)	0.39 + 0.17 (0.2 to 0.8)
*p* value	0.005^*∗*^	0.003^*∗*^	0.003^*∗*^	0.003^*∗*^	0.002^*∗*^
1ry piggyback: CDVA (logMAR)	0.44 + 0.19 (0.1 to 0.8)	0.41 + 0.16 (0.1 to 0.8)	0.42 + 0.17 (0.1 to 0.8)	0.46 + 0.17 (0.2 to 0.8)	0.41 + 0.16 (0.1 to 0.8)
2ry Artisan: CDVA (logMAR)	0.42 + 0.20 (0.2 to 0.7)	0.40 + 0.18 (0.2 to 0.7)	0.39 + 0.16 (0.1 to 0.7)	0.38 + 0.17 (0.1 to 0.7)	0.39 + 0.16 (0.1 to 0.7)
*p* value	0.647	0.728	0.612	0.493	0.653
1ry piggyback: MRSE (D)	1.11 + 1.32 (−1.0 to + 3.5)	1.01 + 1.26 (−1.0 to + 2.75)	1.05 + 1.28 (−1.0 to + 3.0)	1.03 + 1.28 (−1.0 to + 3.0)	1.05 + 1.29 (−1.0 to + 3.0)
2ry Artisan: MRSE (D)	0.12 + 0.72 (−1.0 to +1.5)	0.09 + 0.69 (−1.0 to +1.5)	0.08 + 0.66 (−1.0 to +1.5)	0.08 + 0.67 (−1.0 to +1.5)	0.07 + 0.66 (−1.0 to +1.5)
*p* value	0.024^*∗*^	0.029^*∗*^	0.037^*∗*^	0.039^*∗*^	0.034^*∗*^
1ry piggyback: MAE (D)	1.42 + 1.06 (0.50 to 3.35)	1.39 + 0.93 (0.25 to 3.35)	1.37 + 0.95 (0.25 to 3.10)	1.36 + 0.98 (0.25 to 3.10)	1.35 + 0.96 (0.25 to 3.10)
2ry Artisan: MAE (D)	0.55 + 0.46 (0.25 to 1.15)	0.52 + 0.43 (0.25 to 1.40)	0.51 + 0.40 (0.00 to 1.40)	0.53 + 0.41 (0.00 to 1.40)	0.52 + 0.42 (0.00 to 1.40)
*p* value	0.009^*∗*^	0.007^*∗*^	0.005^*∗*^	0.006^*∗*^	0.005^*∗*^

SD: standard deviation; UDVA: uncorrected distance visual acuity; CDVA: corrected distance visual acuity; MAE: mean absolute error, *p* value: compares mean of 1ry Piggyback vs. 2ry Artisan using independent sample *t*-test. ^*∗*^Statistically significant.

## Data Availability

The data used to support the findings of this study are available from the corresponding author upon request.

## Abbreviations

IOL: Intraocular lens
RLE: Refractive lens exchange
ACD: Anterior chamber depth
UDVA: Uncorrected distance visual acuity
CDVA: Corrected distance visual acuity
AL: Axial length
MRSE: Manifest refraction spherical equivalent
CCT: Central corneal thickness
MAE: Mean absolute error
ILO: Interlenticular opacification.

## References

[1] Park S. H., Ahn Y. J., Shin S. Y., Lee Y. C. (2016). Clinical features of posterior microphthalmos associated with papillomacular fold and high hyperopia. *Clinical and Experimental Optometry*.

[2] Patel N., Khan A. O., Alsahli S. (2018). Genetic investigation of 93 families with microphthalmia or posterior microphthalmos. *Clinical Genetics*.

[3] Nowilaty S. R., Khan A. O., Aldahmesh M. A., Tabbara K. F., Al-Amri A., Alkuraya F. S. (2013). Biometric and molecular characterization of clinically diagnosed posterior microphthalmos. *American Journal of Ophthalmology*.

[4] Relhan N., Jalali S., Pehre N., L Rao H., Manusani U., Bodduluri L. (2015). High-hyperopia database, part I: clinical characterisation including morphometric (biometric) differentiation of posterior microphthalmos from nanophthalmos. *Eye*.

[5] Khairallah M., Messaoud R., Zaouali S., Yahia S. B., Ladjimi A., Jenzri S. (2002). Posterior segment changes associated with posterior microphthalmos. *Ophthalmology*.

[6] Lemos J. A., Rodrigues P., Resende R. A., Menezes C., Gonçalves R. S., Coelho P. (2015). Cataract surgery in patients with nanophthalmos: results and complications. *European Journal of Ophthalmology*.

[7] Carifi G., Pitsas C., Zygoura V., Kopsachilis N. (2013). Cataract surgery in patients with nanophthalmos. *Ophthalmology*.

[8] Wladis E. J., Gewirtz M. B., Guo S. (2006). Cataract surgery in the small adult eye. *Survey of Ophthalmology*.

[9] Hoffer K. J. (2000). Clinical results using the Holladay 2 intraocular lens power formula. *Journal of Cataract & Refractive Surgery*.

[10] Aristodemou P., Knox Cartwright N. E., Sparrow J. M., Johnston R. L. (2011). Formula choice: Hoffer Q, Holladay 1, or SRK/T and refractive outcomes in 8108 eyes after cataract surgery with biometry by partial coherence interferometry. *Journal of Cataract & Refractive Surgery*.

[11] Gayton J. L., Sanders V. N. (1993). Implanting two posterior chamber intraocular lenses in a case of microphthalmos. *Journal of Cataract & Refractive Surgery*.

[12] Hua X., Yuan X. Y., Song H., Tang X. (2013). Long-term results of clear lens extraction combined with piggyback intraocular lens implantation to correct high hyperopia. *International journal of ophthalmology*.

[13] Cao K. Y., Sit M., Braga-Mele R. (2007). Primary piggyback implantation of 3 intraocular lenses in nanophthalmos. *Journal of Cataract & Refractive Surgery*.

[14] Kohnen T. (2018). Iris-fixated phakic intraocular lenses: new results. *Ophthalmology*.

[15] Xu W., Ye P. P., Yao K. (2011). Correction of extreme hyperopia: artisan iris-fixated intraocular lens implantation for pseudophakia after clear lens extraction. *International Journal of Ophthalmology*.

[16] Lifshitz T., Levy J. (2005). Secondary artisan phakic intraocular lens for correction of progressive high myopia in a pseudophakic child. *Journal of American Association for Pediatric Ophthalmology and Strabismus*.

[17] Reinstein D. Z., Waring G. O. (2009). Graphic reporting of outcomes of refractive surgery. *Journal of Refractive Surgery*.

[18] Reinstein D. Z., Archer T. J., Srinivasan S. (2017). Standard for reporting refractive outcomes of intraocular lens-based refractive surgery. *Journal of Refractive Surgery*.

[19] Alexander P., Matheson D., Baxter J., Tint N. L. (2012). United Kingdom national cataract training survey. *Journal of Cataract & Refractive Surgery*.

[20] Hoffer K. J. (2015). Accuracy of the refractive prediction determined by multiple currently available intraocular lens power calculation formulas in small eyes. *American Journal of Ophthalmology*.

[21] Holladay J. T., Gills J. P., Leidlein J., Cherchio M. (1996). Achieving emmetropia in extremely short eyes with two piggyback posterior chamber intraocular lenses. *Ophthalmology*.

[22] De Silva S. R., Arun K., Anandan M., Glover N., Patel C. K., Rosen P. (2011). Iris-claw intraocular lenses to correct aphakia in the absence of capsule support. *Journal of Cataract & Refractive Surgery*.

[23] Steijns D., Bijlsma W. R., Van der Lelij A. (2013). Cataract surgery in patients with nanophthalmos. *Ophthalmology*.

